# A high-throughput and ultrasensitive identification methodology for unauthorized GMP component based on suspension array and logical calculator

**DOI:** 10.1038/s41598-019-43863-7

**Published:** 2019-05-13

**Authors:** Pengyu Zhu, Wei Fu, Shuang Wei, Xiao Liu, Chenguang Wang, Yun Lu, Ying Shang, Xiyang Wu, Yuping Wu, Shuifang Zhu

**Affiliations:** 1 Chinese Academy of Inspection and Quarantine, Beijing, 100029 China; 2 Guangdong Entry-Exit Inspection and Quarantine Bureau, Guangdong, 510000 China; 3 Yunnan Insititute of Food Safety, Kunmming University of Science and technology, Yunnan, 650500 China; 4 Department of Food Science and Engineering, Jinan University, Guangzhou, 510000 China

**Keywords:** Nucleic acids, High-throughput screening, Transgenic plants

## Abstract

To solve the problem of the unauthorized GMP components within import and export goods, the LI-US (Logic Identification of unauthorized GMP content by Universal-primer Suspension-array) system, which takes advantage of suspension array and logic calculator, was developed in the present study. Seventeen signal input channels have been optimized and validated in our research to ensure the multiplex practicality of the LI-US system. Three LI-US logic gates, including a YES gate, an OR gate and an AND gate, were designed as different detection strategies for GMP identification. The feasibility and specificity of the LI-US system were validated in the present study. Combining the optimization and evaluation of the signal input procedure, the sensitivity of this LI-US system reached 0.05% of the GMP mass concentration. The practicability evaluation of LI-US demonstrated its application within different substrates and varieties. In conclusion, the LI-US system was developed with extremely high specificity, sensitivity and practicability among different substrates and varieties, which could meet the demands of unauthorized GMP contents for both import and export goods.

## Introduction

Since the first commercialized event, the delayed-maturity tomatoes FLAVR SAVR™ in 1996, the industry of genetically modified plants (GMPs) has achieved significant development^1^. According to the statistical data of the ISAAA, the plant hectares of GMPs have reached 189.8 million in 2016, an increase of 2.5% compared to that obtained in 2016^2^. The number of commercialized GMP events, which covers most of the plant varieties, including the maize, soybean and canola, has reached 476 within 67 countries^2^. However, the explosive development of the GMP industry has drawn the attention of governments because of the uncertainty of GMP safety and the unbalanced development of different countries^3–5^. For most countries and areas, the authorization of GMP events could be an extremely strict and complicated procedure involving the distinct evaluation of both the human and environments of different countries. The transmission of GMP events that have not been authorized in one country would pose a severe risk to the human population and environment. Therefore, different countries and areas have declared various labeling laws to regulate inner- and inter-country GMP circulation^6^. For instance, the European Union (EU) has declared a threshold of 0.9% for authorized GMP content, while the threshold of unauthorized GMP content is 0.1%. In 2016, the China Inspection Quarantine detected an unauthorized MIR162 event among maize goods imported from America^7^. All goods that contained the MIR162 event were rejected and caused an economic loss of millions of dollars. Thus, the establishment of unauthorized GMP component identification methodology is essential to regulate the commercialization of the GMP industry.

As the identification of unauthorized GMP components includes the PCR amplification of different screening elements or event sequences^8–11^, the utilization of high-throughput technologies is essential to save labor and achieve reliable results. Recently, several technologies, including ChIP-^12,13^, sequencing-^8,14^ and PCR-based methodologies^15,16^, have been developed to meet various demands, such as gene expression evaluation, SNP identification and multiplex component distinction. Among the different technologies, PCR-based methods, particularly multiplex PCR, are the most easily operated methods with an extremely low threshold for both facilities and operators^15,16^. However, these methods would be not convenient due to the increase in target amplicons. The optimized multiplex amplification methods, such as the ME-qPCR and UP-PCR, have been developed to overcome this drawback. The ME-qPCR is firstly mentioned in 2005 with the definition of MT-PCR. This technology have been optimized and utilized for the area of GMP components by Fu^16^ and Wei^17^. Based on the combination of universal primer^18^, the UP-ME-qPCR has been developed to ensure the multiplex amplification more stable and feasible. For technologies based on sequencing, different studies have reported the identification of unknown sequences^8,14,19,20^. However, higher facility costs and required operator experience have limited the spread of these technologies. ChIP-based methodologies could achieve the best high-throughput identification among all technologies^12,13^. Traditional ChIP-based methods are not always convenient for large facilities due to the man-made chips and expensive experimental costs. Therefore, suspension array has been developed to make ChIP-based methods more convenient and feasible.

Suspension array, known as liquid bead array, has been developed to achieve extremely high-throughput identification through advanced fluidics, optics and digital signal processing, combining polystyrene microsphere sets for the detection of multiplex targets^21–25^. Taking advantage of different fluorescence dyes, polystyrene microspheres are divided into hundreds of distinct sets to achieve the simultaneous detection of hundreds of components^21^. Therefore, this technique has been widely used in different areas, including the identification of biology markers^23,25^ and the detection of multiple components and toxins^26–28^. Compared to other detection methodologies, this technology can simultaneously generate an extremely high amount of data. Therefore, data processing methods followed by suspension array are important for establishing a high-throughput detection system.

Traditional data processing methodologies and facilities are primarily based on silicon semiconductor-based electronic computers or other packages. However, since the development of data processing, the limitations of traditional methods have been observed due to the computing and storage capacity on a miniaturized scale^29–31^. Thus, the implementation of molecular computing has been proposed as an alternative to achieve biological information processing^32^. As the basic unit of molecular computing, different logic gates have drawn the attention of different researchers to process different biological data based on distinct biological signal “inputs” and “outputs”^33–37^. Due to the demand of conformational polymorphism, molecular recognition ability, specific signal identification and physicochemical stability, DNA is regarded as the most convenient basis for the establishment of molecular calculators. During the past decade, researchers have established and optimized variable DNA logic gates, including YES, NOT, AND, OR, NAND, NOR, XOR, XNOR, INHIBIT, half adder, and half-subtractor^33,35,36^, based on certain valuable DNA features for the interaction of both living and nonliving matter. Because of the comparably higher sensitivity and specificity and lower experimental costs, the fluorescence signal has become the most popular and efficient method to establish different logic gates.

To identify unauthorized GMP components within an extremely short period, a high-throughput identification system, the LI-US (Logic Identification of Unauthorized GMP content by Universal-primer Suspension-array) system, was developed based on the nucleic acid amplification of UP-ME-qPCR, signal identification of suspension array and data processing of logic gates. Due to different practical situations, different logic gates, including the YES gate, the OR gate and the AND gate, were evaluated in the present study. According to the final results, all logic calculator basics developed herein could identify GMP components under different conditions. The sensitivity of this LI-US system was evaluated as 0.05% of the GMP mass concentration.

## Materials and Methods

### GMP samples

Nearly 40 GMP events, involving most of the main plant varieties, such as maize, soybean, canola, etc., were utilized in the present study. Different GMP events were kindly provided by the developers (details listed in Table S1).

### Preparation of experimental samples

To evaluate different experimental parameters, the original samples were serially diluted with non-GMPs to prepare samples with mass concentrations of 50%, 10%, 5%, 1%, 0.5%, 0.1%, 0.05% and 0.01%. All positive and negative GMP kernels were ground using a Retsch® MM430 mixer to achieve a balanced concentration. To avoid heat damage to the plant genomes, the grounding procedure included 10 cycles of agitation for 20 s and cooling to room temperature for 1 min. Subsequently, positive and negative GMP powders were mixed according to the different mass concentrations to generate the raw experimental samples. All raw samples were placed on a Dynamic CM-200 mixer and shaken overnight for equal distribution. The genomic samples were prepared using the CTAB method^38^.

### Primer design

To improve the sensitivity and stability of the suspension array detection system, UP-ME-qPCR (Universal Primer Multiplex Enrichment quantitative PCR) was utilized to achieve pre-amplification. The UP sequence in our research is chosen from the previous research^18^. According to the previous study^16^, ME-qPCR is an excellent pre-amplification method for the simultaneous amplification of up to 40 amplicons in a single PCR reaction. To improve the amplification uniformity of different amplicons, we combined the UP method with ME-qPCR. The first-round primers for reference and screening genes were located on the target sequence of the foreign insertion, while the primers for the event genes were located on the border of foreign and endogenous genes. The length of all amplicons of the first-round amplification was approximately 300 bp. The second-round primers, which generate an amplicon of less than 150 bp, were located within the amplicon of the first-round PCR product. The sequences for all primers used in the present study are listed in Table S2. All primers were synthesized by Invitrogen (Life Technologies, Wilmington, DE, USA).

### Probe design

All the probes used in the present study were designed by Primer Express 3 according to the second-round target sequence of each amplicon. All designed probe sequences were initially evaluated using BLAST among the sequence information (data achieved from https://www.ncbi.nlm.nih.gov) of all elements used in the present study. Furthermore, to avoid the inhibitory effect caused by the multiplex oligonucleotides within the suspension array system, all probes were evaluated for potential hybridization and inner-hairpin structures. All probes used in the present study were purified by HPLC and synthesized by Invitrogen. The purified probes were modified by an amino-modified 12-carbon spacer at the 5′ end (Invitrogen). The sequences and Tm values are listed in Table 1.

**Table 1:** Hybridization probes used in the present study.

Target	Sequence (5′-3′)	Tm^a^
P35S	GGGATGACGCACAATCCCACTATCC	65.21
TNOS	GGTCTTGCGATGATTATCATATAATTTCTGTTGAATT	64.94
T35S	CCCTAATTTCCCTTATCGGGAAACTACTCAC	64.68
PAT	TGGCCGCGGTTTGTGATATCGTTAA	65.16
Rbc	ATAAGATTCATGGAATTATCTTCCACGTGGC	64.74
E9	GGTTTTCGCTATCGAACTGTGAAATGGAAATG	65.37
PINII	AGTGATTAGCATGTCACTATGTGTGCATCC	65.21
40278	CGTAGCTAACCTTCATTGTATTCCGCTTCA	65.16
305423	TGACACAAATGATTTTCATACAAAAGTCGAGAAA	64.83
CV127	AGCTGTCCCATGCCCATCAAAGAAG	65.34
MON810	CGACCTGAACGAGGACTTTCGGTAG	64.76
NK603	CCGCGTTAACAAGCTTACTCGAGGT	65.22
Bt11	GGCGGCTTATCTGTCTCAGGGG	64.70
MON88017	TTGCCGGAGTATGACGGTGACG	64.81
GA21	GCAGGTGGGTCCGGGTCG	65.09
MON87427	CGGTCGGGTCAAATGTAGAAAATCGG	65.19
MIR604	AGAAGGCGGGAAACGACAATCTGAT	64.56
UP	TTTGGTCGTGGTGGTGGTTT	60.32

^a^The Tm value for each hybridization probe was calculated through the NCBI primer Blast website (https://blast.ncbi.nlm.nih.gov/Blast.cgi).

### ME-qPCR amplification

The ME-qPCR contained two amplification procedures. For the first-round amplification, a 50 μl reaction volume containing 1× multiplex PCR buffer (TaKaRa, Dalian), 0.1 μmol/L first-round primers for different amplicons, 200 ng of DNA template and ddH_2_O up to the final volume was prepared. The following thermal cycling conditions were used for the first-step PCR: 94 °C for 5 min; followed by 15 cycles at 94 °C for 30 s, 60 °C for 30 s, and 72 °C for 90 s; and a final elongation at 72 °C for 5 min. The first-round amplification product was diluted 100-fold and used as the template for the second-round amplification.

For the second-round amplification, UP-modified sequence-specific primers were used. The 25 μl reaction volume containing 1× multiplex PCR buffer, 0.05 μmol/L UP-modified sequence-specific primers for each amplicon, 0.5 μmol/L UP, 2 μL of 100-time diluted first-round PCR product and ddH_2_O to the final volume was prepared. The following thermal cycling conditions were used for the second-step PCR: 94 °C for 5 min; followed by 40 cycles of 94 °C for 30 s and 60 °C for 2 min; and a final elongation at 72 °C for 5 min. The resulting amplification products for this step were evaluated by suspension array.

### Bead activation and coating of microsphere beads with probes

In the present study, Bio-Plex Pro Magnetic COOH Beads (BioRad, California, USA) were utilized for the suspension array analysis. The beads were covalently coated with amino-labeled probes according to the manufacturer’s instructions. All activation and coating steps were performed in the dark to avoid prolonged light exposure. Prior to the experiment, 1-ethyl-3-[3-dimethylaminopropyl] carbodiimide hydrochloride (EDAC) powder was desiccated at room temperature to achieve equilibration. A 1 mL aliquot of the stock fluid (1.25 × 10^7^ beads) was vortexed for 30 s, followed by sonication for 15 s. The sonic-processed products were transferred to a 1.5 mL microcentrifuge tube (Axygen Scientific, CA, USA), and the tube was placed on a magnetic separator to remove the supernatant. The separated beads were then resuspended in 50 μL of 0.1 M MES buffer (pH 4.5), followed by vortexing and sonication for approximately 30 s. Then, 2.5 μL of freshly prepared 10 mg/mL EDAC was mixed with the sonication products and incubated at room temperature for 30 min. After repeating the last step, 1 mL of 0.02% Tween-20 was added, and the mixture was placed on the magnetic separator for approximately 60 s. The separated beads were then washed 2 times with 1 ml of 0.1% sodium dodecyl sulfate (SDS, Sigma-Aldrich) by vortexing. The probe-coated beads were suspended in 100 μL of TE buffer (pH 8.0), vortexed and then sonicated for approximately 20 s. Finally, the well-prepared beads were stored at 4 °C.

### Hybridization of probe-coated beads and target amplicons

Before the hybridization of the beads and target amplicons, the probe-coated beads were suspended in 1 mL of 1.5× TMAC buffer (1.5 M tetramethylammonium chloride, 75 mM Tris, 6 mM EDTA, and 0.15% Sarkosyl, pH 8.0; Sigma-Aldrich). For the Bio-Plex suspension array, a 50 μL reaction volume containing 33 μL of beads mixture, 5 μL of amplification products and 12 μL of TE buffer was prepared. The reaction was incubated at 94 °C for 5 min, 58 °C for 30 min, and then held at 4 °C.

### Input signal identification

After hybridization, the beads were suspended in 100 μL of SAPE working solution (3 μg/mL streptavidin phycoerythrin in 1× TMAC buffer). The mixture was then incubated for 10 min at 58 °C and analyzed on the Bio-Plex 200 system (Bio-Rad, CA, USA). In the present study, the median fluorescence intensity (MFI) value of each sample was automatically calculated through the Bio-Plex program as the main element to evaluate the final results. The beads that were not coated with probes were used as negative controls to calculate the background signal of the assays. The positive sample was defined as the group with at least 3-fold signal compared to the background groups.

### Output logic construction

By collecting the transmission information for all commercial GMP events, we previously developed a combination screening element to cover all GMP events. Furthermore, to predict potentially authorized events, we reversely analyzed the collected information. To this end, different logic gates, such as the YES, OR and AND gates, were designed in the present study by grouping the bead codes of different screening elements.

## Results

### Principle of the LI-US system

**Figure 1:**
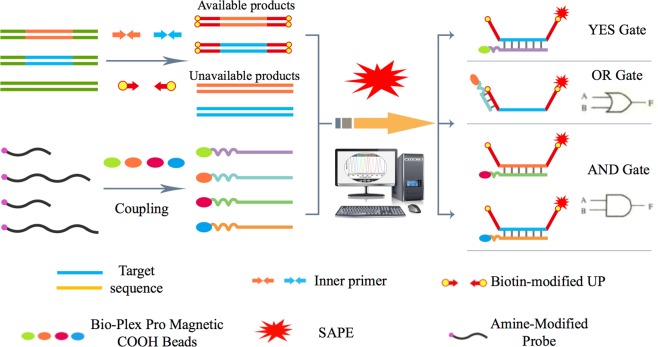
Principle of the LI-US system.

We reported the LI-US system in the present study, which utilizes the suspension array results as the input signal and the presence of the potentially unauthorized GMP content as the output signal. The scheme and principle of the present LI-US system are shown in Fig. 1. The original samples were amplified through two-step ME-qPCR, which included super multiplex first-round amplification and UP-based second-round amplification. The final amplicons for the second step have modified UP sequences compared to their original target sequences. Based on the structure of the amplicons, different logic gates were designed by different hybridization probes or fluorescent types. Thus, the output results varied by the different types of logic gate.

### Optimization and validation of input signal generation

As the sequence screening elements within different GMP events would be variable, the alignment of the screening elements of different GMP events has been firstly performed to ensure the stability and specificity of primer and probes(Fig. S1a). In the present study, the MFI values achieved by the suspension array were regarded as the input signal of the construction of different logic gate systems. The signal input procedure was optimized to ensure the feasibility and sensitivity of the present logic gate system (Fig. S1b). Different elements, including the volume of amplification products (1, 3, 5, 7 and 10 μL), hybridization temperatures (55, 60, 65, 70 and 75 °C) and hybridization duration (10, 20, 30, 50 and 60 min), were evaluated in the present study. The evaluation results are shown in Fig. S1c–e. To optimize the volume of the amplification product, the MFI signal was apparently increased with increasing volume of the amplification product when the volume was relatively low. When this volume exceeded 5 μL, improvements in the MFI value were not observed with increasing volume. Therefore, the final PCR product volume was determined as 5 μL. According to the hybridization temperature and duration optimization, the MFI value was optimal when the temperature was 65 °C and the duration was 30 min.

Moreover, the activated beads coating with probe of different activation duration within 1 month (5 days, 10 days, 15 days, 20 days and 30 days) has been picked to validate the stability. The MFI values of different groups were achieved to analysis the crossing-group difference (Fig. S1f). The final results showed no obvious difference for different groups, indicated that the activated beads coating with probe would be stable for analysis by suspension array within 1 month.

### Specificity validation of input signal

**Figure 2:**
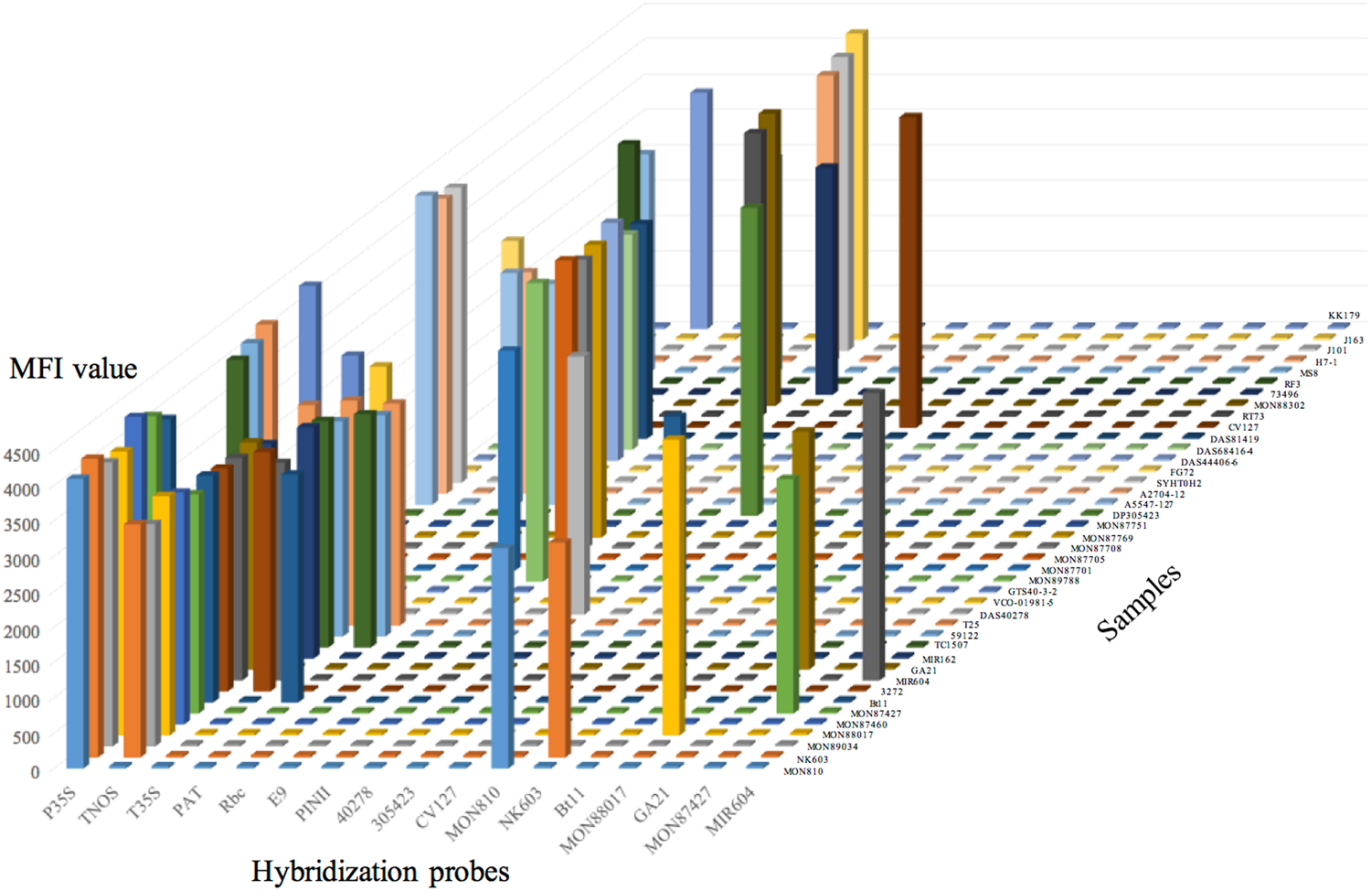
Specificity evaluation of the LI-US system.

To ensure the robustness and practicability of the logic gate system, the specificity of the input signal was first validated. The GMP events among different varieties, including GM maize, soybean, canola, alfalfa and sugar beet, were selected for this experiment. The specificity of the present input signal procedure was validated by the fitness between the experimental results and theoretical screening elements. The validated results of specificity are shown in Fig. 2, and the experimental data are listed in Supplementary Material Table S3. All experimental data were compared with the negative controls with three parallels. According to the validated results, the MFI values of different GMP events were perfectly matched with the theoretical results, indicating that the signal input procedure of the present logic gate system has acceptable specificity among different GMP events.

### Sensitivity validation of input signal

**Figure 3:**
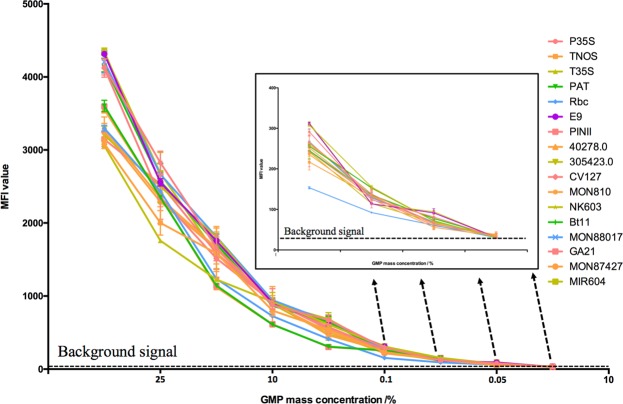
Sensitivity evaluation of the LI-US.

In the present study, the sensitivity was defined as the lowest mass amount and copy number of GMP samples that could be stably detected. GMP samples with different GMP mass contents, including 50%, 10%, 5%, 1%, 0.5%, 0.1%, 0.05% and 0.01%, were generated prior to the validation. The validation results are shown in Fig. 3, and the experimental data are listed in Supplementary Material Table S4. According to the evaluation results, compared to the negative GMP samples, the input signal of all screening elements and reference genes could achieve reliable detection at a mass concentration of 0.05% in the different GMP events. Calculating the combined and genomic sample volume and concentration revealed that the absolute sensitivity of the present signal input procedure could reach a single copy of the target sequence.

### Construction of different logic gates

Based on the sequence structure of the target amplicons, we designed various logic gates for different levels of GMP content detection. The principle and construction of the logic gates in the present study have been comprehensively described below.

#### YES gate

**Figure 4:**
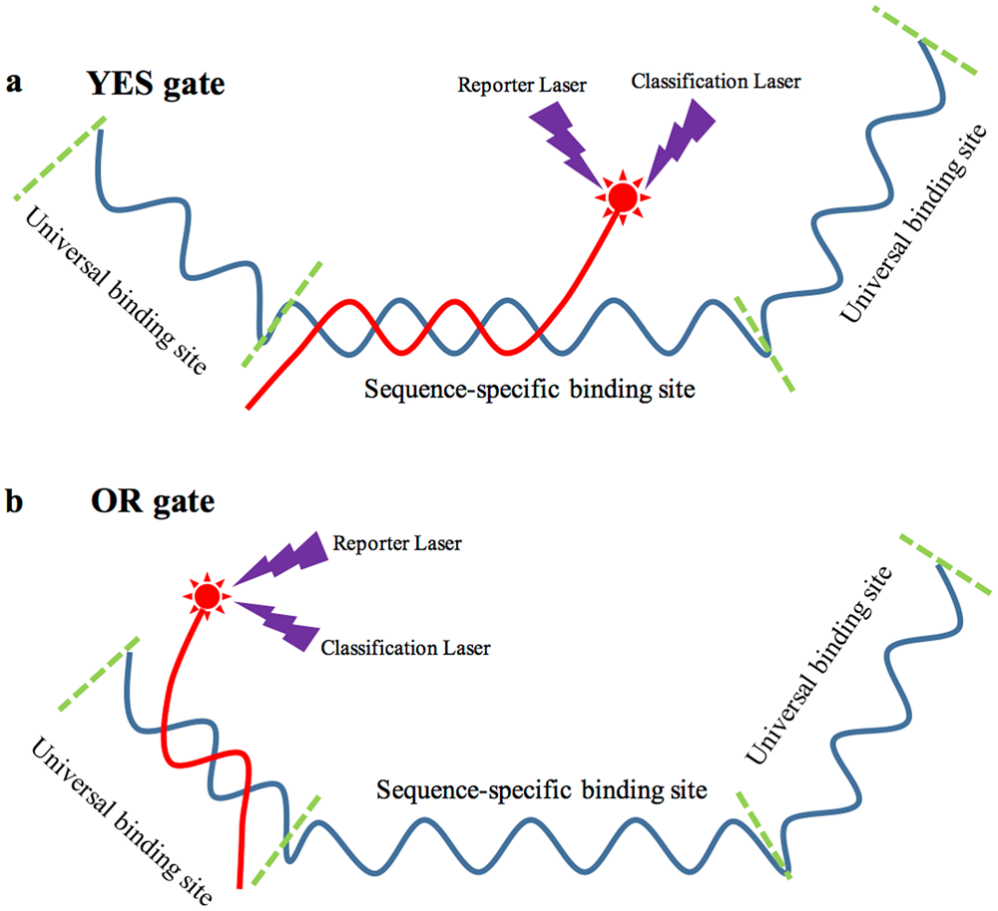
Principle of YES gate and OR gate.

Among all logic gates, the YES gate is the most basic algorithm method. By simply inputting a single signal, the evaluation results would be output according to the intensity and variety of the input signal. For this type of logic gate, the hybridization probes were located at the specific sequence of each amplicon (Fig. 4a). Moreover, the fluorescence types varied for different target sequences. Thus, different screening elements could be easily evaluated. During data processing, the logic gate would generate the FALSE signal (Green) when the input signal of suspension array was 0 or [0, 40], which indicated the no genome sample or negative GMP content within the original reaction volume. When the input signal was between 40 and 150, the YES logic gate would generate the DOUBT signal (Orange), which indicated that extremely low or none GMP content was existed in the reaction volume. The final detection results should be evaluated by the validated methods with higher sensitivity. For the groups generated input signal over 150, TRUE (Red) results would be generated, indicated the mass concentration of GMP events in the original sample was above 0.05%. The relationship between the input signal and the output evaluation results were shown in Supplementary Fig. S2a. For further analysis, the GMP content could be predicted by combining the screening elements.

#### OR gate

Although the YES gate could achieve the explicit judgment for the experimental screening elements, it could be inconvenient when dealing with a large amount of target amplicons. Thus, we further constructed the OR gate to achieve the easy identification of GMP content. The OR gate generates the “TRUE” output results when at least one of the input signals is positive (TRUE or DOUBT). In the present study, the OR gate was used to evaluate the general GMP content present in the sample. For the OR gate, a hybridization probe was designed to target the universal sequence, which was induced by preamplification through ME-PCR (Fig. 4b). During the input data processing of OR gate, the input signal was defined as “1” for the positive result, indicating that the sample contained at least one screening element. The true table between the input signal and output signal is listed in Supplementary Fig. S2b, which was evaluated with a mixture sample of MON810 (authorized GMP component) and GA21 (unauthorized GMP component) events. According to the results, the samples that contained at least one GMP event output the TRUE signal, indicating the fitness between the experimental and theoretical results.

#### AND gate

In the present study, we constructed the AND gate to identify potentially unauthorized GMP components only if both input signal channels were “1”. To this end, two input signal channels, including the positive input signal for theoretical screening elements and the reversed input signal for other screening elements, were designed. The positive input signal would generate “1” when the theoretical screening elements generated positive evaluation results, while the reversed group would generate “0” when other screening elements showed positive results. For the positive or reversed input signal, the screening elements of each group were modified with unique fluorescent labels that could easily be recognized during data processing. According to the definition of the AND gate, the output results would be TRUE unless both channels had input signals of “1”, indicating that no potentially unauthorized GMP components were detected within the target sample. To evaluate the practicability of the AND gate, different GMP events, MON810 (authorized component), NK603 and Bt11, were selected for evaluation. The grouped screening elements and the logic calculation results are listed in Supplementary Material Fig. S2c. The output results were FALSE when the input signal was (0,0), (1,0) or (0,1), which indicated the abnormality of the target samples. The TRUE results of the AND gate indicated that no unauthorized screening existed in the target samples.

### Real sample evaluation

To further test the feasibility in practical use, the logic gates in the present study were applied to identify unauthorized GMP components. Different GMP samples have been prepared by spiking the positive GMP events with non-GMP matrix bought from the market (Merry Market, Beijing, China). The detailed information and evaluation results of different samples are shown in Table 2. The results were consistent with the results under ideal conditions, which suggested that interference of materials in practical samples would not interact with the signal generation reaction and that this device can be used to analyze practical samples. Using this method, the practical samples could easily be identified to determine whether they contained GMP or unauthorized GMP components.

**Table 2:** Practical evaluation of the LI-US system.

Variety	Matrix	GMP component		Evaluation results by LI-US			Evaluation results by qPCR		Fitness
		Authorized	Unauthorized	YES gate	OR gate	AND gate	Authorized	Unauthorized	
Maize	Maize meal	1% MON810	5% GA21	TRUE	TRUE	FALSE	+	+	YES
			0.5% GA21	TRUE	TRUE	FALSE	+	+	YES
			0.05% GA21	TRUE	TRUE	FALSE	+	+	YES
			No GA21	TRUE	TRUE	TRUE	+	−	YES
Soybean	Soybean meal	1% GTS40-3-2	5% MON89788	TRUE	TRUE	FALSE	+	+	YES
			0.5% MON89788	TRUE	TRUE	FALSE	+	+	YES
			0.05% MON89788	TRUE	TRUE	FALSE	+	?^a^	YES
			No MON89788	TRUE	TRUE	TRUE	+	−	YES
Canola	Canola seeds	1% RT73	5% RF3	TRUE	TRUE	FALSE	+	+	YES
			0.5% RF3	TRUE	TRUE	FALSE	+	+	YES
			0.05% RF3	TRUE	TRUE	FALSE	+	?	YES
			No RF3	TRUE	TRUE	TRUE	+	−	YES

^a^The groups with symbol of “?” indicated the uncertainty of the evaluation results, which have achieved the Ct values above 36.

## Discussion

By combining the signal input of suspension array with calculations by different logic gates, we achieved the LI-US system, a serial GMP content logic detection strategies to meet the different conditions of GMP identification. By optimizing the signal input procedure, the sensitivity achieved a mass concentration of 0.05%, which could meet most demands in daily detection.

In the present study, a screening element group covering all commercial GMP events was used to establish an unauthorized GMP component identification method. This group contained 7 screening elements and 3 unique events that contained no regular screening gene. By simultaneously detecting the above target genes, the existence of GMP contents could be evaluated. Thus, the balanced multiplex amplification of the target amplicon would be extremely essential for the final logic analysis results. To improve the amplification efficiency and equality of different amplicons, we utilized an optimized multiplex amplification method, taking advantage of universal primers and ME-qPCR. In a previous study, we developed an efficient multiplex amplification methodology, ME-qPCR, based on the two-step amplification of three pairs of primers, to achieve the extremely sensitive and multiplex amplification. Moreover, to further reduce the side effects caused by the addition of oligonucleotides, the UP method was combined to balance the amplification efficiency of different amplicons. By combining the UP method, balanced amplification products were generated, leading to a distinct MFI value during the suspension array step. The UP structure was also essential for the logic analysis of the final input signal. As all the amplification products were modified with an UP sequence, the identification of this structure would be convenient for the rapid detection of the GMP content. Thus, the UP structure would be necessary for not only balanced multiplex amplification but also the diversity of different logic output results.

In the present study, three basic logic gates, YES, OR and AND gates, were developed for the identification of screening elements, GMP components and unauthorized GMP components, respectively. This logic device could easily achieve GMP evaluation results by inputting the signal generated by the suspension array. For practical detection, the identification of GMP contents could be divided into three parts: the detection of different screening elements, the detection of the general GMP contents and the identification of potential unauthorized GMP components. Thus, the three logic gates in the present study would be perfectly matched for practical detection. The evaluation results of the real samples also confirmed the practicability of the present logic system. However, the logic gates developed in the present study could only distinguish the unauthorized GMP component that contained different screening elements compared to the theoretical GMP events. For instance, when the authorized GMP component was declared as NK603 events, which contained both CaMV35S promoter and NOS terminator, the potential unauthorized GMP component that only contained one or two elements of the CaMV35S promoter and NOS terminator would be impossible to identify. Therefore, utilizing the high-throughput detection of suspension array, this GMP event was further evaluated. To precisely identify the potential unauthorized GMP events containing fewer or the same number of screening elements, the hybridization probes for these events were added to the negative signal groups of the AND gates. For NK603, MON810, GA21, MON88017, MON87427 and MIR604 events contained only the screening elements of the CaMV35S promoter and NOS terminator. Therefore, to make the logic analysis results more convincing, we modified the AND logic gates by adding the potential unauthorized GMP events to the reversed signal group. The comparison of improved and previous AND gates is shown in Supplementary Fig. S3a,b. Taking the authorized GMP event NK603 for instance, the original reversed signal group contains T35S, PAT, Rbc, E9, PINII, 40278, 305423 and CV127 genes, while the improved reversed signal input group contains five more genes, MON810, GA21, MON88017, MON87427 and MIR604. The original AND gates would generate the false TRUE result while the unauthorized GMP components contain the same or less screening elements compared with the authorized GMP. Although this step would generate an additional experimental workload, the precise evaluation results would be generated with much lower experimental load than the other normal methodologies. After this optimization step, the logic device developed in the present study was validated to have excellent stability and practicability.

## Conclusion

The LI-US system based on a logic calculator for the identification of unauthorized GMP component was developed utilizing the input signal generation by UP-ME-qPCR and suspension array. Through the optimization and validation of the signal input procedure and logic gates, we achieved the stable identification of unauthorized GMP components in three strategies with a mass concentration as low as 0.05%. (1) The LI-US system could be regarded as a cost-efficient detection methods for the identification of unauthorized GMP contents (Table S5). (2) The YES gate is the most basic calculation method for the rapid identification of different screening elements. (3) The OR gate is used to identify the GMP content within the target sample, which could generate TRUE results when at least one screening element was positive. (4) The AND gate is used to identify the unauthorized GMP components through a combination of different screening elements. The validation results of both specificity and real samples also ensured the practicability of the present logic calculation system. Moreover, considering the emerging of the new GMP events, this logic system could easily be expanded by adding new screening elements or GMP events.

## Supplementary information

Supplementary Material
