# Supplementary material for: A high-throughput and ultrasensitive identification methodology for unauthorized GMP component based on suspension array and logical calculator
Source: Sci Rep. 2019 May 13;9:7311. doi: 10.1038/s41598-019-43863-7 (PMC6513989; doi:10.1038/s41598-019-43863-7)
Supplement: Supplementary file 1 — Supplementary Material [file 41598_2019_43863_MOESM1_ESM.docx]

A high-throughput and ultrasensitive identification methodology for unauthorized GMP component based on suspension array and logical calculator

Pengyu Zhu^1a^, Wei Fu^1a^, Shuang Wei^2^, Xiao Liu^1^, Chenguang Wang^1^, Yun Lu^1^, Ying Shang^3^, Xiyang Wu^4^, Yuping Wu^1*^, Shuifang Zhu^1*^

^1^Chinese Academy of Inspection and Quarantine, Beijing, 100029, China

^2^Guangdong Entry-Exit Inspection and Quarantine Bureau, Guangdong, 510000, China

^3^Yunnan Insititute of Food Safety, Kunmming University of Science and technology, Yunnan 650500, China

^4^Department of Food Science and Engineering, Jinan University, Guangzhou, China

* To whom correspondence should be addressed.

^a^ These authors contribute equally to this work.

Tel/Fax: +86 010 53897568; Email: [wuyuping@caiqtest.com](mailto:wuyuping@caiqtest.com), zhusf@caiq.org.cn

Running title: GMP identification based on suspension array and logical calculator

Number of tables: 2

Number of figures: 5

No figure in color.


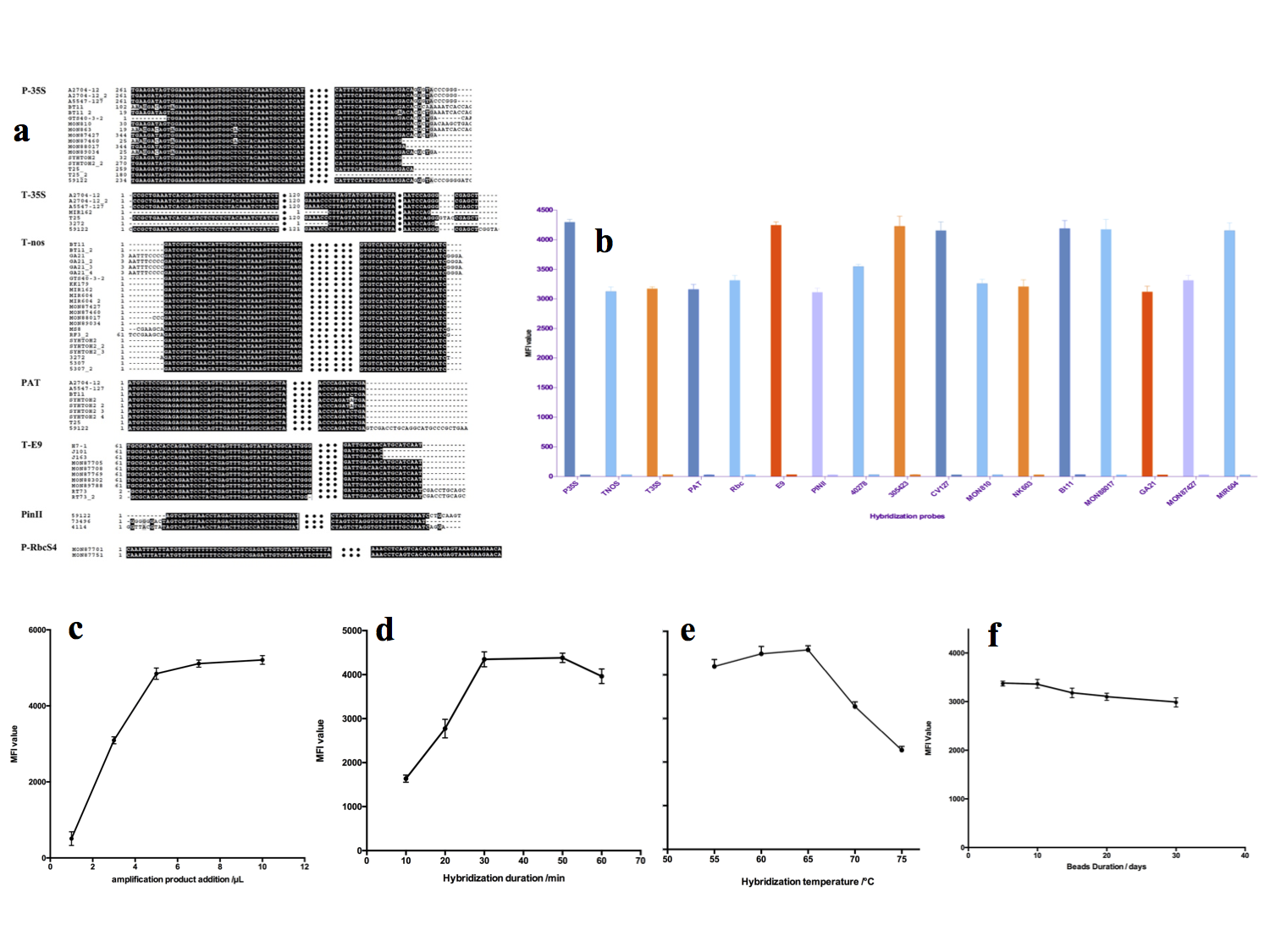


Supplementary Figure S1 Optimization and validation of signal input procedure of LI-US

a: Sequence alignment of screening elements in different GMO events

b: Application evaluation of input signal of LI-US system;

c-e: Optimization of the volume of amplification products, hybridization temperatures and hybridization duration, respectively.

f: Duration stability of activated beads coating with probes


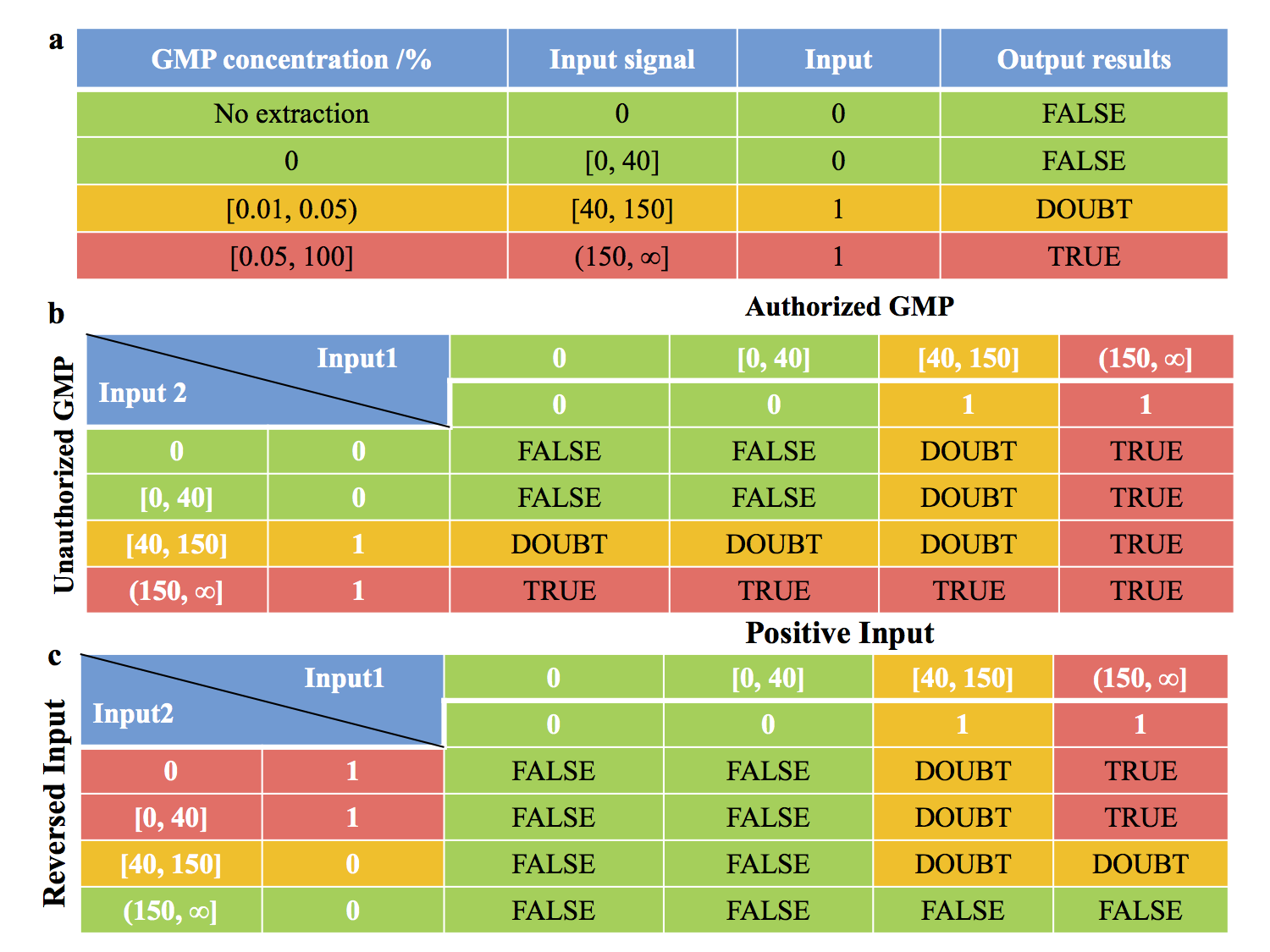


Supplementary Figure S2 True table of YES, OR and AND gates of the LI-US system.

a: YES gate; b: OR gate; c: AND gate.

The output signal would be TRUE only if the input signal of both positive and reversed groups were “1”.


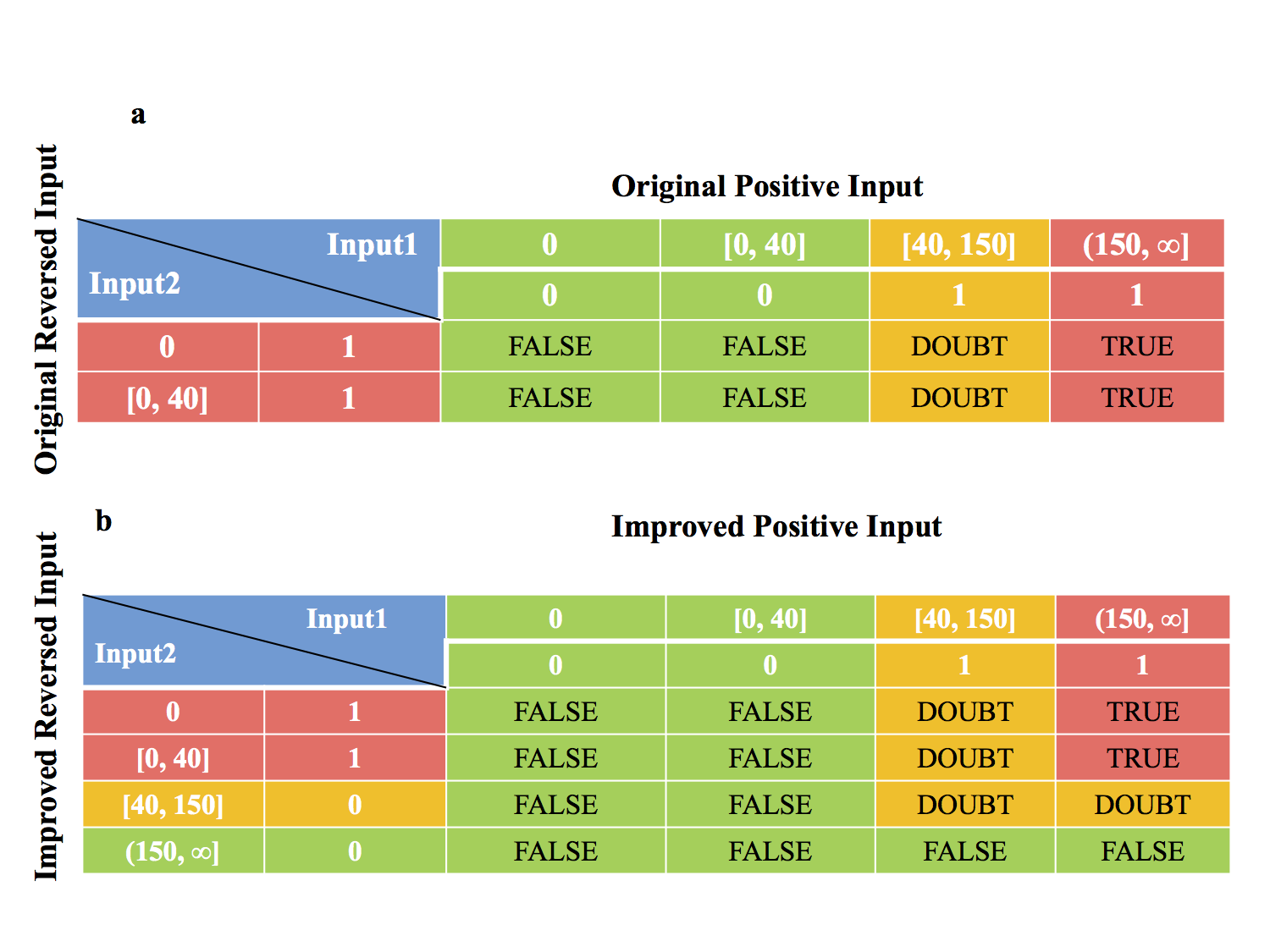


Supplementary Figure S3 Original (a) and improved (b) version of true table of AND gate of the LI-US system.

Supplementary Table S1 The details of GMP events used in our research

| GMP events | Variety | Developer | Screening Elements |
| --- | --- | --- | --- |
| MON810 | Maize | Monsanto | P35S |
| NK603 |  | Monsanto | P35S, TNOS |
| MON89034 |  | Monsanto | P35S, TNOS |
| MON88017 |  | Monsanto | P35S, TNOS |
| MON87460 |  | Monsanto | P35S, TNOS |
| MON87427 |  | Monsanto | P35S, TNOS |
| Bt11 |  | Syngenta | P35S, TNOS, PAT |
| 3272 |  | Syngenta | T35S, TNOS |
| MIR604 |  | Syngenta | TNOS |
| GA21 |  | Syngenta | TNOS |
| MIR162 |  | Syngenta | T35S, TNOS |
| TC1507 |  | Dupont | P35S, T35S, PAT |
| 59122 |  | Dupont | P35S, T35S, PAT |
| T25 |  | Bayer | P35S, T35S, PAT |
| DAS40278 |  | Dows | None |
| VCO-01981-5 |  | Genective S.A. | T35S |
| GTS40-3-2 | Soybean | Monsanto | P35S, TNOS |
| MON89788 |  | Monsanto | E9 |
| MON87701 |  | Monsanto | Rbc |
| MON87705 |  | Monsanto | E9 |
| MON87708 |  | Monsanto | E9 |
| MON87769 |  | Monsanto | E9 |
| MON87751 |  | Monsanto | Rbc |
| DP305423 |  | Dupont | None |
| A5547-127 |  | Bayer | P35S, T35S, PAT |
| A2704-12 |  | Bayer | P35S, T35S, PAT |
| SYHT0H2 |  | Syngenta | P35S, PAT |
| FG72 |  | Bayer | TNOS |
| DAS44406-6 |  | Dows | PAT |
| DAS68416-4 |  | Dows | PAT |
| DAS81419 |  | Dows | PAT |
| CV127 |  | BASF | None |
| RT73(GT73) | Canola | Monsanto | E9 |
| MON88302 |  | Monsanto | E9 |
| 73496 |  | Dupont | PINII |
| RF3 |  | Bayer | TNOS, Rbc |
| MS8 |  | Bayer | TNOS, Rbc |
| H7-1 | Sugarbeet | Monsanto | E9 |
| J101 | Alfalfa | Monsanto | E9 |
| J163 |  | Monsanto | E9 |
| KK179 |  | Monsanto | TNOS |

The detailed information of the GMP events has been listed in this table. The P35S is short for the 35S promoter, TNOS for NOS terminator, T35S for 35S terminator, NPTII for Neomycin Phosphotransferase II.

Supplementary Table S2 Amplification primers used in our research

| Target | Primer type | Forward primer | Reverse primer | Amplicon length |
| --- | --- | --- | --- | --- |
| P35S | Outer-P | ATTGATGTGATATCTCCACTGACGT | CCTCTCCAAATGAAATGAACTTCCT | 101 |
|  | Inner-P | TTTGGTCGTGGTGGTGGTTTTCTCCACTGACGTAAGGGATGA | TTTGGTCGTGGTGGTGGTTTGAAGGGTCTTGCGAAGGATAG | 95^1^ |
| TNOS | Outer-P | TGCCGGTCTTGCGATGA | AAATGTATAATTGCGGGACTCTAATC | 128 |
|  | Inner-P | TTTGGTCGTGGTGGTGGTTTTGCCGGTCTTGCGATGA | TTTGGTCGTGGTGGTGGTTTCCCATCTCATAAATAACGTCATGC | 135 |
| T35S | Outer-P | GGGGTTTCTTATATGCTCAACACATG | TCACCAGTCTCTCTCTACAAATCTATCAC | 130 |
|  | Inner-P | TTTGGTCGTGGTGGTGGTTTCGAAACCCTATAAGAACCCTAAT | TTTGGTCGTGGTGGTGGTTTGTCTCTCTCTACAAATCTATCATCTC | 136 |
| PAT | Outer-P | GTCGACATGTCTCCGGAGAG | GCAACCAACCAAGGGTATC | 191 |
|  | Inner-P | TTTGGTCGTGGTGGTGGTTTAGAGGAGACCAGTTGAGATTA | TTTGGTCGTGGTGGTGGTTTGTGGCTCTGTCCTAAAGTTC | 151 |
| Rbc | Outer-P | CCACTCCACCATCACACAATTTC | GGAGAGGTGTTGAGACCCTTATC | 112 |
|  | Inner-P | TTTGGTCGTGGTGGTGGTTTCCATCACACAATTTCACTCATAG | TTTGGTCGTGGTGGTGGTTTGCTTGAACCGCTGGAATA | 120 |
| E9 | Outer-P | TCTTGTACCATTTGTTGTGCTTGT | GGACCATATCATTCATTAACTCTTCTCC | 108 |
|  | Inner-P | TTTGGTCGTGGTGGTGGTTTGTTGTGCTTGTAATTTACTGTG | TTTGGTCGTGGTGGTGGTTTCTCCATCCATTTCCATTTCAC | 111 |
| PINII | Outer-P | GACTTGTCCATCTTCTGGATTGG | CACACAACTTTGATGCCCACAT | 105 |
|  | Inner-P | TTTGGTCGTGGTGGTGGTTTTCTGGATTGGCCAACTTAAT | TTTGGTCGTGGTGGTGGTTTCAACTTTGATGCCCACATTAT | 128 |
| 40278 | Outer-P | CACGAACCATTGAGTTACAATC | TGGTTCATTGTATTCTGGCTTTG | 98 |
|  | Inner-P | TTTGGTCGTGGTGGTGGTTTCATTGAGTTACAATCAACAGCAC | TTTGGTCGTGGTGGTGGTTTTCGTAGCTAACCTTCATTGTATTC | 101 |
| 305423 | Outer-P | CGTGTTCTCTTTTTGGCTAGC | GTGACCAATGAATACATAACACAAACTA | 93 |
|  | Inner-P | TTTGGTCGTGGTGGTGGTTTTTGGCTAGCTAGTGTTTT | TTTGGTCGTGGTGGTGGTTTACTATTGACACAAATGAT | 97 |
| CV127 | Outer-P | CGTTGAGCTTTAAGACGTTTGG | GTGGCCCTTCTCGCCTTAT | 105 |
|  | Inner-P | TTTGGTCGTGGTGGTGGTTTCCCATGCCCATCAAAGAA | TTTGGTCGTGGTGGTGGTTTGCCCTTCTCGCCTTATTG | 111 |
| MON810 | Outer-P | AACGTGCCCGGTACTGGTTC | GACTGCTCGCAAGCAAATTC | 167 |
|  | Inner-P | TTTGGTCGTGGTGGTGGTTTCCACAGCCACCACTTCTCC | TTTGGTCGTGGTGGTGGTTTGCAAGCAAATTCGGAAATGAA | 134 |
| NK603 | Outer-P | CGGCCAGCAAGCCTTGTAG | TTTGGACTATCCCGACTCTCTTC | 120 |
|  | Inner-P | TTTGGTCGTGGTGGTGGTTTGGCCAGCAAGCCTTGTA | TTTGGTCGTGGTGGTGGTTTGACTATCCCGACTCTCTTCTCA | 157 |
| BT11 | Outer-P | CTGGGAGGCCAAGGTATCTAAT | GCTGCTGTAGCTGGCCTAATCT | 189 |
|  | Inner-P | TTTGGTCGTGGTGGTGGTTTCCTTCTTGGCGGCTTATCT | TTTGGTCGTGGTGGTGGTTTCATGTCGAGATCCGAGGGA | 101 |
| Mon88017 | Outer-P | AGCAGCAGAATCGTGTGACAAC | TTTCCCGGACATGAAGCCAT | 120 |
|  | Inner-P | TTTGGTCGTGGTGGTGGTTTCACATCATCGACAAGCACCTT | TTTGGTCGTGGTGGTGGTTTCGGACATGAAGCCATTTACAAT | 116 |
| GA21 | Outer-P | CGTTATGCTATTTGCAACTTTAGAACA | GCGATCCTCCTCGCGTT | 112 |
|  | Inner-P | TTTGGTCGTGGTGGTGGTTTCTCTTTCTCAACAGCAGGT | TTTGGTCGTGGTGGTGGTTTGATCCTCCTCGCGTTTC | 97 |
| Mon87427 | Outer-P | ACGGAATAAGTCCTCTCCCG | CCATATTGACCATCATACTCATTGC | 176 |
|  | Inner-P | TTTGGTCGTGGTGGTGGTTTACGGAAACGGTCGGGTCAAATG | TTTGGTCGTGGTGGTGGTTTCCATGTAGATTTCCCGGTTTTCTC | 135 |
| MIR604 | Outer-P | GGCTGGACGCCAGATCAC | GCCGTTTTACGTTTGGAACTG | 178 |
|  | Inner-P | TTTGGTCGTGGTGGTGGTTTGCGCACGCAATTCAACAG | TTTGGTCGTGGTGGTGGTTTGGTCATAACGTGACTCCCTTAATTCT | 116 |
| Universal Primer (UP) | | TTTGGTCGTGGTGGTGGTTT | | 40 |

1: The amplicon length of inner primers was summed by the inner primers and UP sequence.

Supplementary Table S3 Specificity validation results in our research

|  | P35S | TNOS | T35S | PAT | Rbc | E9 | PINII | 40278 | 305423 | CV127 | MON810 | NK603 | Bt11 | MON88017 | GA21 | MON87427 | MIR604 |
| --- | --- | --- | --- | --- | --- | --- | --- | --- | --- | --- | --- | --- | --- | --- | --- | --- | --- |
| MON810 | 4096.4 | 33.4 | 33.1 | 32.8 | 32.3 | 30.6 | 30.6 | 31.1 | 30.6 | 31.9 | 3116.1 | 34.0 | 33.8 | 30.3 | 31.5 | 33.7 | 33.4 |
| NK603 | 4225.8 | 3296.9 | 31.9 | 30.1 | 33.2 | 30.1 | 31.9 | 33.9 | 32.5 | 31.3 | 30.8 | 3039.8 | 32.4 | 33.8 | 31.3 | 30.7 | 31.1 |
| MON89034 | 4018.5 | 3146.3 | 31.8 | 30.6 | 31.9 | 30.8 | 31.2 | 30.4 | 33.2 | 31.6 | 32.9 | 31.3 | 33.3 | 32.2 | 31.2 | 32.5 | 30.1 |
| MON88017 | 4017.0 | 3385.4 | 32.6 | 32.6 | 30.6 | 30.2 | 32.8 | 31.8 | 33.7 | 30.5 | 33.0 | 30.5 | 32.3 | 4182.8 | 32.9 | 30.5 | 33.7 |
| MON87460 | 4349.0 | 3280.8 | 31.9 | 33.9 | 32.0 | 34.0 | 30.1 | 31.1 | 31.4 | 34.0 | 33.2 | 33.7 | 30.8 | 33.6 | 33.3 | 31.6 | 32.9 |
| MON87427 | 4207.3 | 3100.2 | 33.5 | 31.3 | 32.2 | 31.9 | 32.2 | 32.8 | 32.2 | 33.6 | 32.2 | 33.0 | 31.9 | 31.0 | 30.4 | 3316.1 | 30.9 |
| Bt11 | 4008.5 | 3209.3 | 31.3 | 3224.9 | 33.4 | 31.2 | 31.9 | 30.2 | 30.8 | 30.7 | 32.2 | 30.2 | 4043.7 | 30.2 | 32.6 | 33.3 | 31.9 |
| 3272 | 32.4 | 3154.1 | 3384.5 | 32.1 | 31.3 | 32.6 | 33.7 | 33.8 | 30.3 | 30.5 | 30.7 | 31.7 | 30.7 | 32.4 | 30.6 | 31.5 | 33.9 |
| MIR604 | 31.6 | 3146.8 | 3078.7 | 30.5 | 30.5 | 30.7 | 33.4 | 30.6 | 30.5 | 33.3 | 32.2 | 32.1 | 34.0 | 33.9 | 32.0 | 30.8 | 4066.9 |
| GA21 | 30.1 | 3212.8 | 33.7 | 31.9 | 30.8 | 33.8 | 33.4 | 33.6 | 31.2 | 31.5 | 30.3 | 31.5 | 33.4 | 30.1 | 3367.9 | 30.5 | 33.3 |
| MIR162 | 30.5 | 3034.3 | 3271.1 | 31.1 | 32.9 | 32.7 | 31.6 | 33.4 | 33.6 | 30.7 | 31.5 | 30.8 | 33.7 | 32.0 | 31.9 | 32.3 | 30.2 |
| TC1507 | 4070.8 | 31.3 | 3206.2 | 3301.6 | 33.3 | 33.1 | 31.1 | 30.2 | 32.9 | 31.5 | 31.1 | 31.1 | 30.3 | 31.4 | 30.1 | 33.3 | 32.5 |
| 59122 | 4148.7 | 31.2 | 3044.4 | 3132.6 | 33.1 | 31.4 | 30.4 | 32.5 | 30.2 | 33.1 | 31.5 | 31.6 | 32.3 | 31.0 | 30.2 | 32.3 | 32.2 |
| T25 | 4259.9 | 3118.9 | 3182.3 | 3141.0 | 32.2 | 31.0 | 30.6 | 33.2 | 33.3 | 32.3 | 30.5 | 33.4 | 30.4 | 33.3 | 31.0 | 33.1 | 30.1 |
| DAS40278 | 33.2 | 32.4 | 30.9 | 31.8 | 33.2 | 30.8 | 30.6 | 3652.4 | 30.6 | 30.1 | 32.8 | 32.8 | 33.4 | 32.3 | 33.7 | 31.5 | 31.5 |
| VCO-01981-5 | 32.3 | 33.8 | 3354.4 | 31.3 | 32.2 | 32.1 | 30.9 | 31.7 | 30.1 | 33.5 | 33.9 | 32.1 | 32.7 | 30.2 | 32.1 | 32.1 | 30.6 |
| GTS40-3-2 | 4340.4 | 3352.8 | 32.9 | 33.4 | 31.6 | 32.0 | 30.2 | 30.1 | 33.7 | 32.6 | 32.2 | 30.6 | 31.6 | 30.0 | 30.3 | 32.2 | 31.0 |
| MON89788 | 31.4 | 31.7 | 31.5 | 34.0 | 31.8 | 4224.9 | 32.3 | 31.6 | 31.0 | 31.4 | 32.9 | 31.7 | 32.4 | 30.3 | 32.2 | 33.7 | 31.3 |
| MON87701 | 32.2 | 32.9 | 31.4 | 30.6 | 3112.7 | 32.7 | 31.7 | 30.1 | 32.7 | 31.5 | 30.4 | 31.4 | 32.0 | 32.9 | 33.0 | 31.2 | 32.4 |
| MON87705 | 32.2 | 33.0 | 33.4 | 31.9 | 32.0 | 4233.9 | 31.5 | 31.2 | 30.3 | 32.4 | 32.5 | 32.4 | 30.5 | 32.0 | 33.1 | 31.2 | 32.5 |
| MON87708 | 33.8 | 32.1 | 32.4 | 33.2 | 33.0 | 4088.5 | 33.9 | 33.5 | 31.8 | 33.9 | 30.8 | 31.7 | 31.8 | 31.5 | 31.5 | 33.6 | 32.6 |
| MON87769 | 31.9 | 30.7 | 30.4 | 33.1 | 31.8 | 4141.8 | 33.9 | 33.9 | 31.9 | 33.5 | 32.4 | 34.0 | 33.7 | 30.9 | 30.6 | 33.6 | 32.6 |
| MON87751 | 32.2 | 32.5 | 32.6 | 33.3 | 3109.6 | 33.2 | 30.5 | 30.4 | 31.4 | 33.9 | 31.0 | 33.6 | 33.4 | 32.6 | 32.6 | 33.7 | 31.3 |
| DP305423 | 33.8 | 31.1 | 31.4 | 33.2 | 30.8 | 33.4 | 31.4 | 32.2 | 4355.5 | 30.8 | 31.9 | 31.0 | 31.5 | 30.9 | 30.8 | 31.9 | 31.3 |
| A5547-127 | 4372.6 | 31.4 | 3277.3 | 3121.4 | 32.2 | 31.9 | 32.1 | 31.8 | 32.6 | 33.4 | 30.5 | 30.7 | 30.0 | 30.4 | 31.0 | 30.9 | 34.0 |
| A2704-12 | 4171.2 | 30.6 | 3134.6 | 3118.3 | 31.6 | 32.1 | 32.8 | 32.8 | 30.8 | 32.8 | 32.6 | 32.7 | 31.9 | 33.1 | 32.2 | 31.9 | 30.6 |
| SYHT0H2 | 4174.0 | 32.6 | 33.9 | 3043.3 | 30.8 | 32.4 | 32.4 | 31.9 | 33.3 | 30.2 | 31.4 | 30.8 | 32.2 | 31.0 | 32.3 | 30.4 | 31.0 |
| FG72 | 33.0 | 3265.4 | 33.5 | 31.3 | 32.6 | 32.0 | 32.6 | 33.2 | 30.0 | 32.6 | 32.7 | 31.6 | 34.0 | 32.4 | 32.1 | 33.9 | 33.4 |
| DAS44406-6 | 33.4 | 31.0 | 33.9 | 3365.6 | 33.7 | 31.6 | 33.9 | 32.4 | 30.3 | 33.2 | 32.8 | 30.8 | 31.6 | 30.9 | 33.3 | 30.7 | 32.8 |
| DAS68416-4 | 31.7 | 33.2 | 33.1 | 3044.3 | 33.3 | 32.4 | 30.4 | 33.7 | 31.8 | 34.0 | 33.4 | 31.6 | 33.2 | 32.2 | 33.8 | 30.4 | 32.6 |
| DAS81419 | 33.8 | 31.3 | 32.4 | 3038.7 | 31.6 | 33.5 | 32.5 | 33.6 | 31.4 | 33.7 | 31.0 | 32.0 | 33.0 | 31.5 | 30.5 | 30.4 | 30.8 |
| CV127 | 31.8 | 31.9 | 31.4 | 31.5 | 33.6 | 33.7 | 32.9 | 33.2 | 33.2 | 4394.5 | 30.2 | 32.2 | 32.4 | 30.2 | 30.7 | 32.4 | 30.3 |
| RT73(GT73) | 31.4 | 30.3 | 30.4 | 31.3 | 32.6 | 4011.2 | 32.1 | 32.5 | 33.0 | 31.0 | 31.8 | 31.5 | 32.0 | 33.6 | 33.5 | 31.1 | 33.5 |
| MON88302 | 30.3 | 30.2 | 31.3 | 32.7 | 30.7 | 4133.6 | 30.2 | 31.7 | 32.3 | 32.8 | 31.9 | 33.0 | 32.0 | 30.5 | 33.8 | 30.4 | 32.4 |
| 73496 | 32.7 | 32.3 | 33.9 | 33.4 | 32.9 | 31.2 | 3214.5 | 33.3 | 33.8 | 32.8 | 30.7 | 32.8 | 33.9 | 32.8 | 30.7 | 32.0 | 32.7 |
| RF3 | 33.8 | 3389.9 | 33.5 | 30.8 | 3040.7 | 31.2 | 30.9 | 32.2 | 30.7 | 30.3 | 30.5 | 33.7 | 31.1 | 30.1 | 32.7 | 31.5 | 33.4 |
| MS8 | 30.9 | 3092.2 | 30.8 | 30.9 | 3032.4 | 31.0 | 30.1 | 30.2 | 30.9 | 33.0 | 30.9 | 30.6 | 33.3 | 32.3 | 31.2 | 33.9 | 32.2 |
| H7-1 | 30.7 | 33.4 | 30.7 | 30.1 | 30.5 | 4052.7 | 32.4 | 33.4 | 33.3 | 32.4 | 31.6 | 32.6 | 33.6 | 31.7 | 32.6 | 30.1 | 31.8 |
| J101 | 31.1 | 32.3 | 30.6 | 30.7 | 30.2 | 4157.1 | 30.8 | 30.7 | 31.2 | 30.7 | 31.8 | 33.1 | 31.6 | 32.8 | 30.3 | 31.0 | 33.5 |
| J163 | 30.2 | 32.8 | 33.2 | 30.3 | 32.8 | 4332.6 | 30.9 | 33.2 | 32.9 | 31.9 | 32.8 | 33.0 | 33.8 | 30.4 | 33.6 | 31.7 | 31.3 |
| KK179 | 33.0 | 3342.3 | 33.3 | 30.9 | 33.2 | 33.6 | 31.3 | 30.6 | 33.4 | 33.6 | 31.0 | 34.0 | 33.1 | 33.0 | 31.4 | 30.4 | 30.9 |

Supplementary Table S4 Sensitivity validation results in our research

|  | P35S | | | TNOS | | | T35S | | | PAT | | | Rbc | | | E9 | | |
| --- | --- | --- | --- | --- | --- | --- | --- | --- | --- | --- | --- | --- | --- | --- | --- | --- | --- | --- |
|  | 1 | 2 | 3 | 1 | 2 | 3 | 1 | 2 | 3 | 1 | 2 | 3 | 1 | 2 | 3 | 1 | 2 | 3 |
| 50% | 4209.4 | 4011.0 | 4194.5 | 3052.3 | 3044.2 | 3163.8 | 3031.7 | 3059.8 | 3079.6 | 3516.7 | 3665.8 | 3630.2 | 3280.1 | 3344.3 | 3272.9 | 4325.2 | 4303.6 | 4318.3 |
| 10% | 2643.4 | 2914.1 | 2920.1 | 2068.5 | 1810.9 | 2128.2 | 1765.6 | 1771.1 | 1737.4 | 2336.6 | 2346.4 | 2369.5 | 2438.2 | 2445.5 | 2419.0 | 2501.5 | 2576.5 | 2596.5 |
| 5% | 1843.2 | 1666.8 | 1480.3 | 1235.4 | 1595.5 | 1926.3 | 1223.1 | 1248.1 | 1210.3 | 1134.5 | 1146.9 | 1149.2 | 1236.6 | 1243.8 | 1224.5 | 1765.7 | 1743.9 | 1761.3 |
| 1% | 902.9 | 805.2 | 920.4 | 718.4 | 836.1 | 850.6 | 929.4 | 933.1 | 903.8 | 604.0 | 608.1 | 617.5 | 731.2 | 714.6 | 729.6 | 923.0 | 905.1 | 901.3 |
| 0.5% | 595.1 | 711.2 | 716.0 | 562.7 | 459.4 | 598.4 | 616.3 | 606.2 | 610.0 | 309.7 | 306.7 | 308.7 | 416.7 | 413.0 | 411.5 | 615.6 | 615.7 | 604.1 |
| 0.1% | 295.2 | 282.5 | 296.7 | 213.3 | 235.8 | 257.3 | 306.0 | 311.8 | 306.4 | 259.8 | 252.9 | 254.9 | 151.4 | 152.3 | 156.7 | 313.3 | 313.6 | 306.6 |
| 0.05% | 141.4 | 101.9 | 127.9 | 117.7 | 118.6 | 140.4 | 150.5 | 157.3 | 157.5 | 156.1 | 152.6 | 151.4 | 93.3 | 90.3 | 93.1 | 114.8 | 113.2 | 113.2 |
| 0.01% | 69.7 | 80.4 | 94.2 | 62.9 | 53.7 | 51.0 | 70.7 | 71.4 | 70.7 | 72.7 | 70.5 | 72.7 | 60.3 | 61.7 | 61.0 | 99.9 | 79.7 | 94.3 |
| NTC | 30.2 | 34.0 | 31.2 | 33.4 | 37.4 | 32.0 | 30.1 | 31.5 | 30.4 | 30.2 | 30.0 | 30.0 | 30.0 | 31.1 | 30.2 | 31.3 | 30.6 | 31.4 |

|  | PINII | | | 40278 | | | 305423 | | | CV127 | | | MON810 | | | NK603 | | |
| --- | --- | --- | --- | --- | --- | --- | --- | --- | --- | --- | --- | --- | --- | --- | --- | --- | --- | --- |
|  | 1 | 2 | 3 | 1 | 2 | 3 | 1 | 2 | 3 | 1 | 2 | 3 | 1 | 2 | 3 | 1 | 2 | 3 |
| 50% | 3592.5 | 3570.2 | 3628.6 | 3565.6 | 3502.1 | 3592.9 | 4373.9 | 4315.9 | 4356.6 | 4012.5 | 4096.7 | 4258.6 | 3374.3 | 3395.3 | 3098.3 | 3218.6 | 3316.3 | 3071.8 |
| 10% | 2358.2 | 2374.2 | 2347.0 | 2405.4 | 2382.7 | 2311.9 | 2535.3 | 2513.9 | 2609.9 | 2594.7 | 2011.6 | 2456.2 | 2313.9 | 2466.3 | 2237.6 | 2685.3 | 2261.0 | 2557.0 |
| 5% | 1133.9 | 1145.2 | 1142.3 | 1108.4 | 1138.0 | 1118.6 | 1710.7 | 1765.2 | 1769.4 | 1269.3 | 1784.3 | 1492.8 | 1665.5 | 1435.9 | 1873.2 | 1780.2 | 1849.0 | 1794.4 |
| 1% | 602.8 | 616.2 | 624.9 | 604.1 | 618.5 | 609.2 | 933.4 | 917.4 | 904.8 | 931.6 | 877.3 | 947.4 | 838.3 | 899.8 | 1014.5 | 739.6 | 1058.3 | 872.1 |
| 0.5% | 303.4 | 303.9 | 302.3 | 311.6 | 310.7 | 300.1 | 629.4 | 606.1 | 609.7 | 519.0 | 689.6 | 520.5 | 446.8 | 517.3 | 432.2 | 567.1 | 433.0 | 427.8 |
| 0.1% | 259.7 | 256.7 | 252.0 | 259.9 | 258.1 | 260.8 | 314.1 | 313.3 | 313.0 | 238.5 | 292.0 | 265.9 | 279.1 | 221.9 | 254.2 | 247.7 | 228.2 | 248.9 |
| 0.05% | 155.2 | 151.4 | 153.4 | 150.5 | 155.2 | 151.2 | 112.8 | 114.7 | 114.0 | 137.0 | 148.1 | 128.0 | 141.9 | 145.5 | 113.6 | 116.5 | 112.8 | 114.4 |
| 0.01% | 73.0 | 70.8 | 72.5 | 70.4 | 70.4 | 72.9 | 97.2 | 95.3 | 90.1 | 72.8 | 51.4 | 72.6 | 74.1 | 51.2 | 64.4 | 67.0 | 66.2 | 70.4 |
| NTC | 30.4 | 31.0 | 30.2 | 31.2 | 30.9 | 31.0 | 31.5 | 31.3 | 31.4 | 34.7 | 32.3 | 30.7 | 43.1 | 36.5 | 33.4 | 40.1 | 39.4 | 30.4 |

|  | Bt11 | | | MON88017 | | | GA21 | | | MON87427 | | | MIR604 | | |
| --- | --- | --- | --- | --- | --- | --- | --- | --- | --- | --- | --- | --- | --- | --- | --- |
|  | 1 | 2 | 3 | 1 | 2 | 3 | 1 | 2 | 3 | 1 | 2 | 3 | 1 | 2 | 3 |
| 50% | 4267.5 | 4119.5 | 4100.5 | 4261.5 | 4323.3 | 4035.8 | 3253.4 | 3126.2 | 3058.4 | 3269.9 | 3119.4 | 3353.1 | 4254.2 | 4336.7 | 4391.4 |
| 10% | 2293.2 | 2681.7 | 2742.4 | 2778.7 | 2722.1 | 2504.3 | 2572.4 | 2401.7 | 2626.1 | 1955.2 | 2389.6 | 2527.3 | 2756.7 | 2366.1 | 2923.0 |
| 5% | 1863.0 | 1684.5 | 1585.6 | 1816.3 | 1744.6 | 1875.7 | 1442.3 | 1476.2 | 1844.1 | 1709.6 | 1914.5 | 1307.9 | 1712.3 | 1666.2 | 1344.8 |
| 1% | 842.6 | 800.1 | 953.4 | 951.6 | 947.6 | 934.8 | 782.5 | 881.8 | 1120.6 | 609.0 | 1028.8 | 1027.2 | 957.3 | 934.4 | 817.9 |
| 0.5% | 589.2 | 736.6 | 600.5 | 572.1 | 654.6 | 739.2 | 473.1 | 597.3 | 574.8 | 479.9 | 463.1 | 587.5 | 726.1 | 746.3 | 585.6 |
| 0.1% | 227.7 | 240.5 | 267.9 | 273.7 | 293.3 | 234.4 | 247.0 | 280.5 | 203.7 | 202.0 | 239.4 | 209.4 | 259.5 | 291.4 | 241.6 |
| 0.05% | 123.0 | 147.0 | 137.5 | 126.4 | 115.5 | 143.8 | 142.3 | 126.7 | 124.9 | 141.8 | 138.5 | 115.1 | 118.8 | 134.5 | 147.6 |
| 0.01% | 81.1 | 72.0 | 78.7 | 62.2 | 76.0 | 67.9 | 56.0 | 53.5 | 69.4 | 71.3 | 60.9 | 74.5 | 77.6 | 69.3 | 88.1 |
| NTC | 33.4 | 33.5 | 32.0 | 31.2 | 34.3 | 35.0 | 31.1 | 44.7 | 38.7 | 38.7 | 33.6 | 33.3 | 32.9 | 33.6 | 31.7 |

Table S5 Experimental cost comparison between LI-US system and conventional validated method

|  | Primer and probes | Amplification reagent | Signal identification | Total |
| --- | --- | --- | --- | --- |
| LI-US system  (17 targets) | 1000RMB×17 targets | 1 tube×20RMB | 1 tube×100RMB | 17120RMB |
| Conventional validated method  (SN/T 1204-2016)  (17 targets) | 1000RMB×17 targets | 17 tube×20RMB | None | 17340RMB |
